# Hysterosalpingography in The Assessment of Congenital Cervical Anomalies

**DOI:** 10.22074/ijfs.2017.4716

**Published:** 2017-02-16

**Authors:** Fatemeh Zafarani, Firoozeh Ahmadi, Gholam Shahrzad

**Affiliations:** Department of Reproductive Imaging, Reproductive Biomedicine Research Center, Royan Institute for Reproductive Biomedicine, Tehran, Iran

**Keywords:** Hysterosalpingography, Congenital, Cervix, Uterine, Anomalies

## Abstract

Cervical abnormalities may be congenital or acquired. Congenital anomalies of the cervix are rarely isolated, and more commonly accompany other uterine anomalies. Various imaging tools have been used in the assessment of Müllerian duct anomalies (MDAs). Currently, magnetic resonance imaging (MRI) is the modality of choice for definitive diagnosis and classification of these MDAs. Hysterosalpingography is a basic tool for evaluation of infertility and allows us to detect a spectrum of anatomical malformations of the utero-cervix in the setting of MDAs. It provides good outlines of the uterine cavity and fallopian tubes, as well as the cervical canal and isthmus. However, hysterosalpingograms (HSG) cannot be performed in patients with isolated congenital maldevelopment (agenesis/disgenesis) of the cervix. This part of pictorial review illustrates the various radiographic appearances of congenital malformations of the utero-cervix with a brief overview of the embryologic features. Accurate diagnosis of such cases is considered essential for optimal treatment and categorization of each anomaly.

## Introduction

Female genital malformations include various forms of developmental and morphological malformations of the vagina, cervix, uterus, adnexa and associated malformations (VCUAM) (1). Genital malformations have an incidence of up to ~7% in the general population (2).

Congenital anomalies of the cervix are rarely isolated, and are more commonly associated with other uterine and vaginal anomalies. Cervical anomalies may manifest as agenesis, dysgenesis, obstruction, abnormal length, inadequate width, and hypertrophy (3); however, complete or partial duplication of the cervix with a normal uterus and an unusual Müllerian anomaly have been reported (4, 5). Cervical atresia usually presents with primary amenorrhea and cyclic abdominal pain. Depending on the type and degree of maldevelopment of the uterine cervix, a woman’s reproductive potential may be adversely affected.

Several imaging tools have been used in the assessment of Müllerian duct anomalies (MDAs). Although magnetic resonance imaging (MRI) is considered the gold standard for definitive diagnosis and classification of genito-urinary anomalies, especially for complex cases, hysterosalpingography is still an important tool in the early evaluation of infertility. Contrast medium which is slowly injected into the uterus through the cervical canal, provides good outlines of the uterine cavity and fallopian tubes, as well as the cervical canal and isthmus (6).

We retrospectively reviewed 38574 hysterosalpingograms (HSGs) performed over a 29-year period (January 1985-December 2013) by one author (G.Sh.). The indications for HSG included infertility, abnormal uterine bleeding, and symptoms related to uterine fibroids. This review illustrates the various radiographic appearances of congenital malformations of the utero-cervix with a brief overview of the embryologic features.

### Embryology

The female genital tract develops from a pair of Müllerian ducts between 6 and 12 weeks of gestation. The process involves three main stages: i. Development of both Müllerian ducts that form the fallopian tubes, uterus, and cervix and upper two thirds of the vagina, whereas failure of this stage results in agenesis/hypoplasia or a unicornuate uterus, ii. Fusion of the lower Müllerian ducts leads to formation of the uterus and cervix while defects in this phase result in a bicornuate uterus, and iii. Canalization and septal resorption of the central septum which results in a single uterine cavity and cervix, whereas failure of this stage leads to a septate or arcuate uterus. Mesonephric or Wolffian ducts play a role as inductors for adequate development, fusion, and resorption of the walls of Müllerian ducts (7). There is controversy over formation of the vagina. Recent studies have stated that the mesonephric ducts together with the Müllerian tubercle form the vagina (7, 8).

By week 20, the process of development is completed. Development of both Müllerian ducts and the urinary tract occurs from a common ridge of the mesoderm; hence, anomalies of the urinary tract are commonly observed in females with genital malformation.

### Classification system

Accurate classification of a female genital tract malformation is necessary to prevent inadequate surgery and achieve optimal treatment. Until now, several classification systems for female genital tract anomalies have been proposed: the American Fertility Society Classification System (currently American Society for Reproductive Medicine, AFS/ASRM) (9); the embryological-clinical classification system of genito-urinary malformations proposed by Acién and Acién (8, 10), the vagina, cervix, uterus, adnexae and associated Malformations system based on the tumor node metastasis (TNM) principle in oncology (1), and the new European Society of Human Reproduction and Embryology (ESHRE) and European Society for Gynaecological Endoscopy (ESGE) classification systems (11). Most of these classification systems seem to be associated with limitations, especially in the diagnosis of unusual and complex malformations (12).

The AFS classification is based mainly on the description of uterine changes and most widely used as the main classification system for its simplicity, friendliness and clinical usefulness. However, this system is associated with limitations in efficient categorization of female genital anomalies. The accompanying malformation (such as duplex vagina), unusual or/and complex malformations especially those in group I (hypoplasia/agenesis) not fit and are described completely in the AFS classification and so often fails to correctly identified and treated (12). In addition, some other cases of utero-vaginal anomalies have been classified in nine subtypes of septate and bicornuate communicating uteri schemes, as suggested by Toaff et al. (13).

The use of embryological-clinical classification of genitor-urinary malformations seems to unify the current embryological and pathogenic concepts and appear to be the most clinically useful (10). Based on the AFS classification scheme, the cervix is classified as follows: IB (under segmental Müllerian duct agenesis or hypoplasia), III (complete non-fusion of MDAs), IV (incomplete fusion of the superior segments of the uterovaginal canal), V (partial or complete non-resorption of the uterovaginal septum), and VII [MDAs related to diethylstilbestrol (DES) exposure in utero].

The accurate diagnosis of such cases is considered essential for optimal treatment and in support of the embryologic concept.

### Cervical agenesis/dysgenesis

Agenesis/dysgenesis of the cervix is rare and usually occurs in association with complete or partial vaginal agenesis (2). It is difficult to diagnose cervical agenesis. Clinical examination has limited diagnostic value and in most cases hysterosalpingography is impossible.

### Utero-cervical anomalies

#### Uterus didelphys

**Fig.1:**
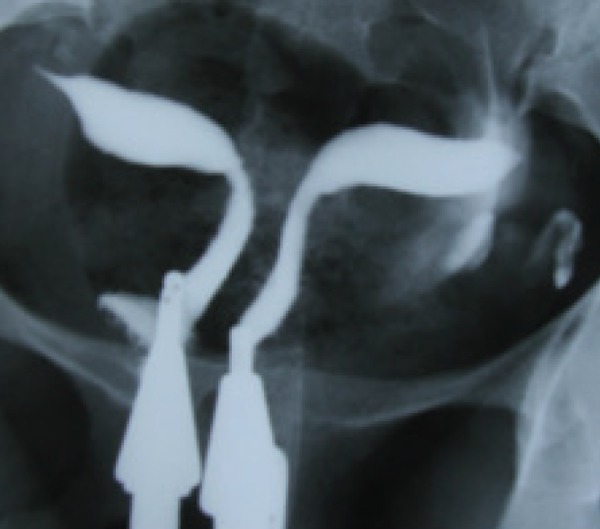
Didelphys uterus in a 31 year-old-woman with 3 years of primary infertility. Hysterosalpingogram demonstrates two symmetrical separate cavities, two cervical canals and presence of double vagina.

Uterus didelphys results from complete failure of Müllerian duct fusion and accounts for approximately 5% of MDAs (2). On hysterosalpingography, two symmetric separate cavities and two cervical canals are present; a double vagina is often present (Fig .1). Didelphys uterus is usually asymptomatic, while cases with unilateral vaginal obstruction may manifest with hematometrocolpos and dysmenorrhea at menarche.

#### Bicornuate uterus

**Fig.2:**
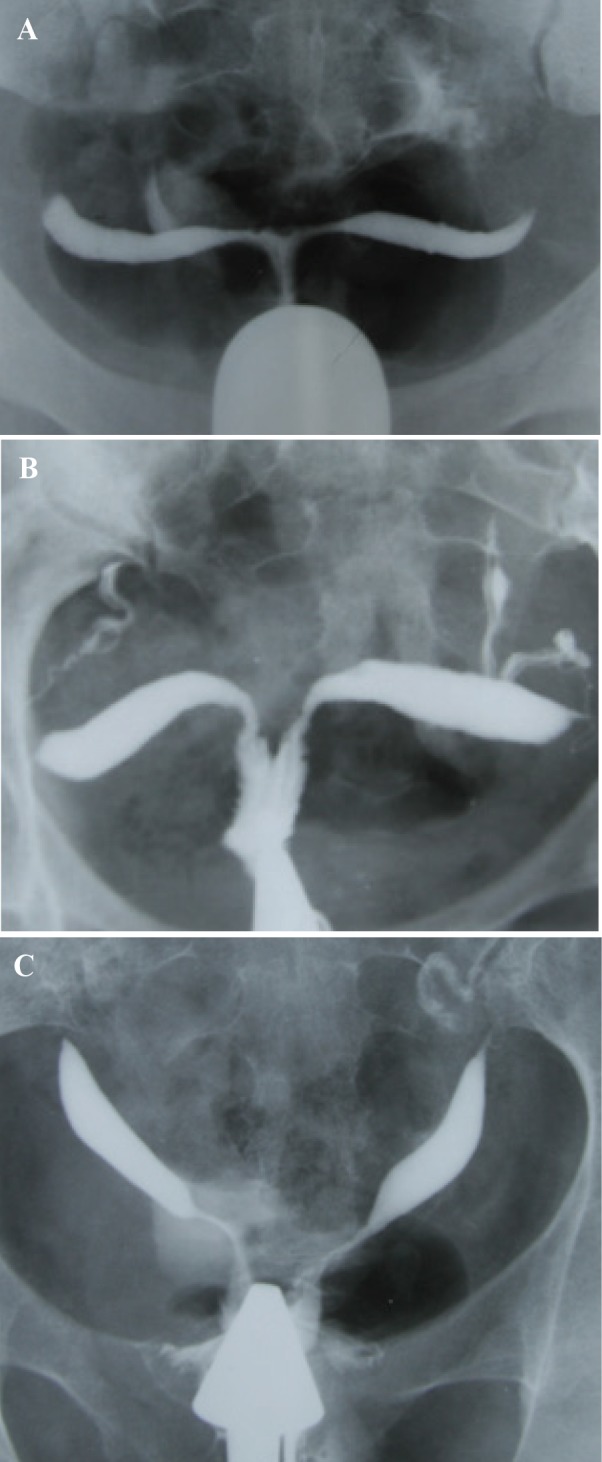
Bicornuate uterus with various degrees of duplication of the cervix in different patients (AFS class IV). A. Bicornuate uterus, consisting of two symmetric uterine cavities with communication at the uterine isthmus, and also intercorneal angle greater than 105º. Single cervix and vagina are present, B. Intervening cleft extends to the endocervical canal, and C. Extension to the level of external os.

The bicornuate uterus represents approximately 25% of MDAs and results from incomplete fusion of the Müllerian ducts at the level of the uterine fundus (2). The two separate uterine cavities are fused caudally and communicate in the lower segment, mostly at the uterine isthmus; a single cervix and vagina are present. Hysterosalpingography demonstrates separate fusiform uterine horns, often with an intercornual angle of >105º (14). There are various degrees of separation between the two horns, as follows: a complete bicornuate uterus, in which the failure to fuse extends the length of the uterine body inferiorly to the internal os; and lesser degrees of a bicornuate uterus, in which the partial interfering cleft is variable in length, extending from the fundus to the cervix (Fig .2).

#### Septate uterus

**Fig.3:**
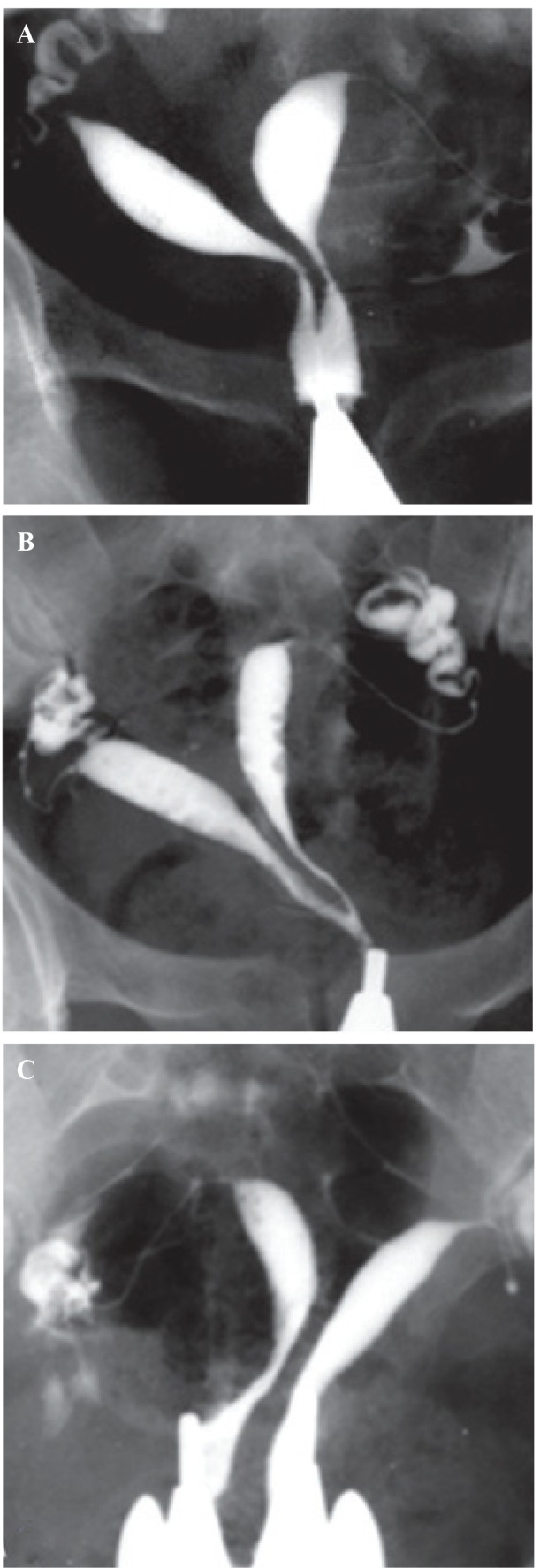
Hysterosalpingography of septate uterus demonstrates a variable degrees of cervical septation in different patients (AFS class V) (12). A. Extension of the midline septum to the lower internal os, B. Septate uterus with extension of septum to the level of external os and production of two cervical canal with one opening, and C. Complete septate uterus with two separate cervical canal (pseudodidelphys).

A septate uterus results from partial or complete failure of resorption of the uterovaginal septum after fusion of the paramesonephric ducts, which occurs in approximately 35% of MDAs (2). Hysterosalpingography of a septate uterus represents varying degrees of the midline septum, extending from the fundus to the cervix and upper vagina, and yielding a V-shaped configuration often with an angle <75º between the two uterine horns (Fig .3). In 25% of cases, complete extension of the septum to the upper vagina is present (Fig .3C) (14).

### Diethylstilbestrol-exposed uterus

Exposure to DES in utero results in multiple, benign abnormalities of the genital tract and clear cell adenocarcinoma of the vagina. DES has been associated with T-shaped and irregular configurations of the endometrial cavity, constrictive bands, structural cervical changes, and cervical anomalies that include hypoplasia, cervical ridges, and cervical collars (15).

**Fig.4:**
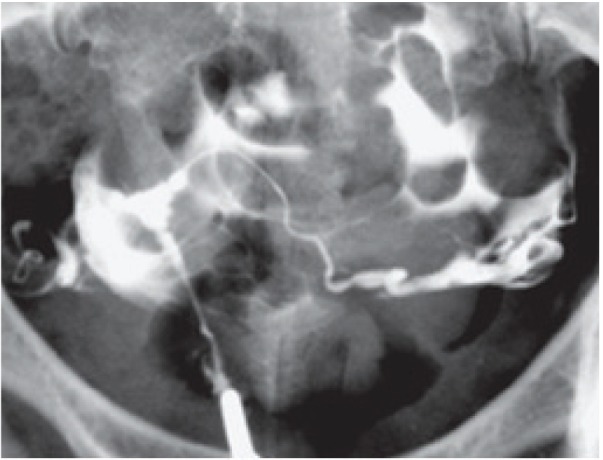
DES-exposure uterus in a 28-year-old infertile patient. Note T- shaped appearance of the endometrial cavity with a long narrowed irregular endocervical canal.

Hysterosalpingography is an excellent screening tool to diagnose DES-related uterine anomalies. The radiographic appearance includes an irregular, narrowed endocervical canal with a shortened upper uterine segment and small, typically irregular cavity that yields a T-shaped uterine configuration (Fig .4).

### Communicating uterus

In 1984, Toaff et al. (13) proposed a classification scheme for nine subtypes of septate and bicornuate uteri that identified the presence of a communication between two separate uterocervical cavities. All types of communicating uteri have an isthmic communication, except for type 9, which has a low cervical communication.

**Fig.5:**
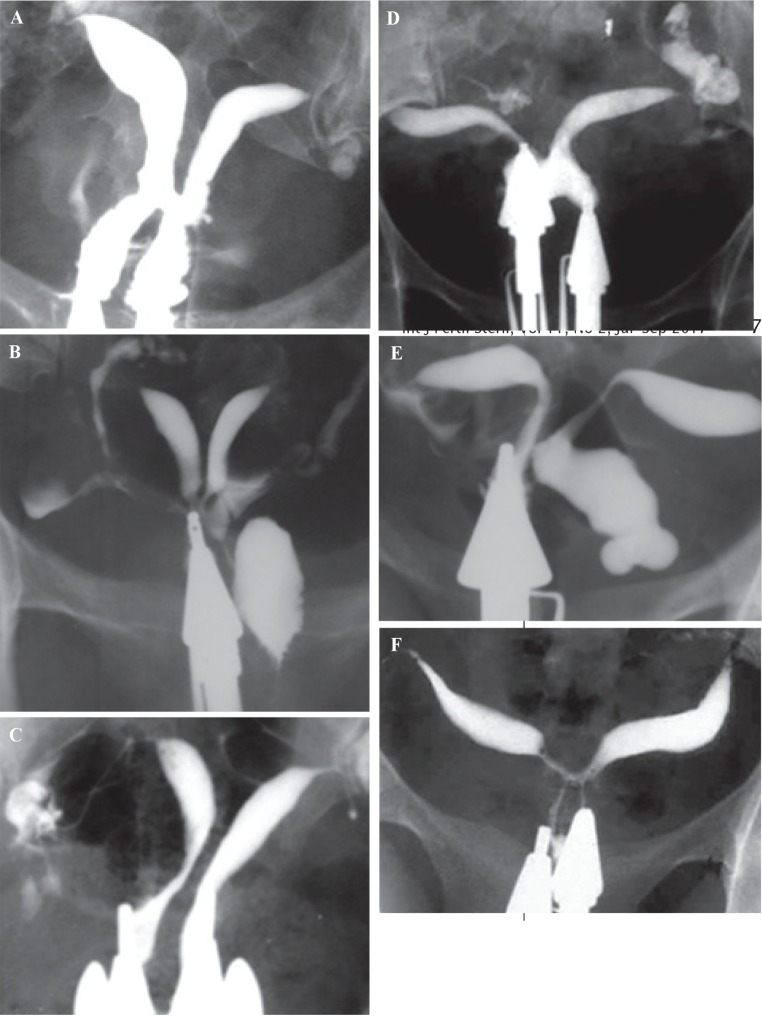
Some cases of communicating uteri with duplication of carvical canal (12). A. Uterus communicants septus, cervix duplex, vagina septa (type 1a). Vaginal septum was observed in vaginal examination, B. Uterus communicants septus, cervix duplex, vagina septa unilateralis atretica (type 2a), C. Uterus communicants septus, cervix septa, vagina septa (type 3a).Vaginal septum was visualized in vaginal examination, D. Uterus comunicants bicornis, cervix duplex, vagina septa (type 4a). Vaginal septum was seen in vaginal examination, E. Uterus communicants bicornis, cervix duplex, vagina septa unilateralis atretica (type 5a), and F. Uterus communicants bicornis, cervix septa, vagina simplex (type 6).

**Fig.6:**
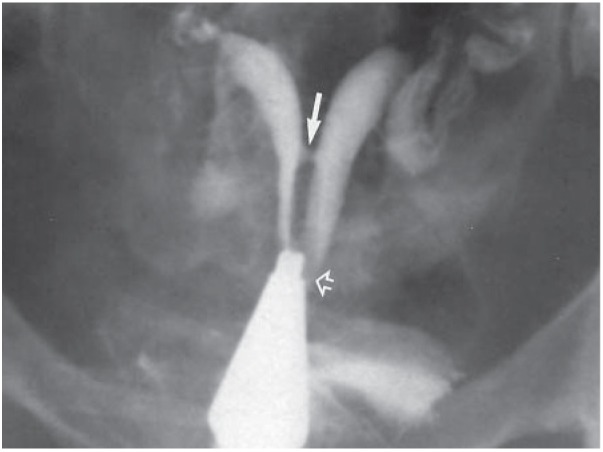
Hysterosalpingography in a 23 year-old-woman with 5 years of primary infertility. The uterus is septate with a midcorporeal communication (straight arrow). The cervix is partially septate and the cervical septum ends a few millimeters above the single external os (open arrow) (16).

**Fig.7:**
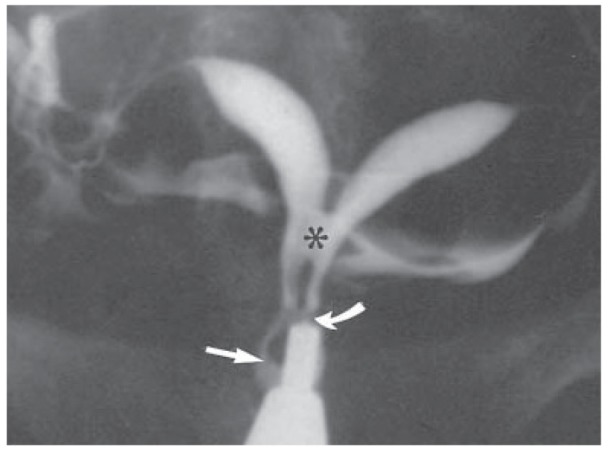
Bicornuate uterus in a 26 year-old-woman with 3 years of primary infertility. Hysterosalpingogram shows the bicornuate uterus with two sites of communication between two uterocervical canal, midcervical (curved arrow) and isthmic (asterisk); septum of the cervix ends several millimeters above the single external os (straight arrow) (16).

**Fig.8:**
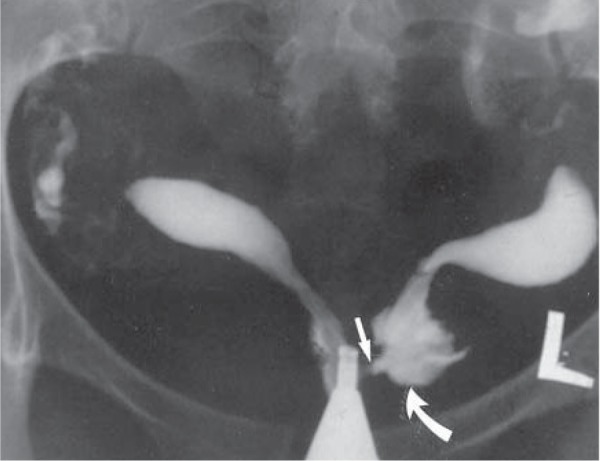
Hysterosalpingography in a 27-year-old woman with primary infertility of 5 years. The uterus is bicornuate with a low cervical communication and left atretic hemicervix. The cannula is located in the right hemicervix. The right external os is visible. Injection of contrast into the right hemicervix was noted to opacify the left hemiuterus and cervix through the low cervical communication (arrow) (16).

During >29 years of one author’s experience (G.SH) in performing HSGs, some rare cases of communicating uteri have been observed and reported (Fig .5). Of these, three represented communications at unusual sites. They were identified as non-isthmic communicating uteri and classified as a new subclass of type 9 (16). The first case was a complete septate uterus with a mid-corporeal communication, in which the septum ended inferiorly several millimeters above the external os (Fig .6). Another case was a bicornuate uterus with two sites of communication, at the mid-cervical and isthmic levels (Fig .7), and a third case with communication that involved the bicornuate uterus with a low cervical communication, left cervico-vaginal atresia, and left renal agenesis (Fig .8).

### Unusual cervical anomaly

**Fig.9:**
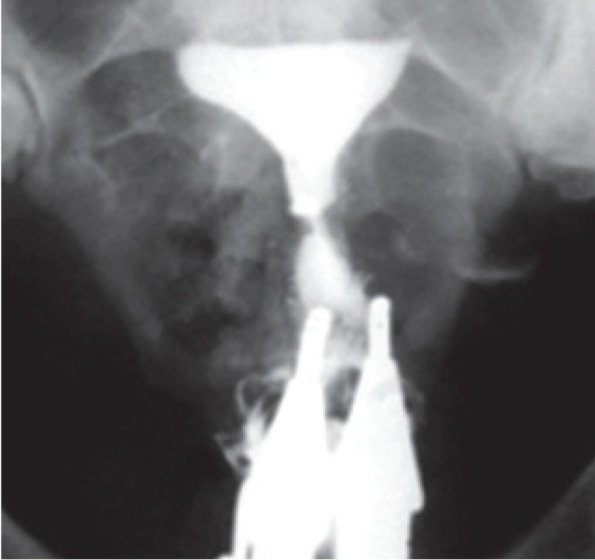
Hysterosalpingogram of a 36 year-old woman shows an normal uterus with septate cervix and vagina.

Some unclassified cases of normal or septate uteri that communicate with double cervices with or without vaginal septa have been previously described (4, 17-19). We encountered a case of normal uterus with septate cervix and vagina (Fig .9).

**Fig.10:**
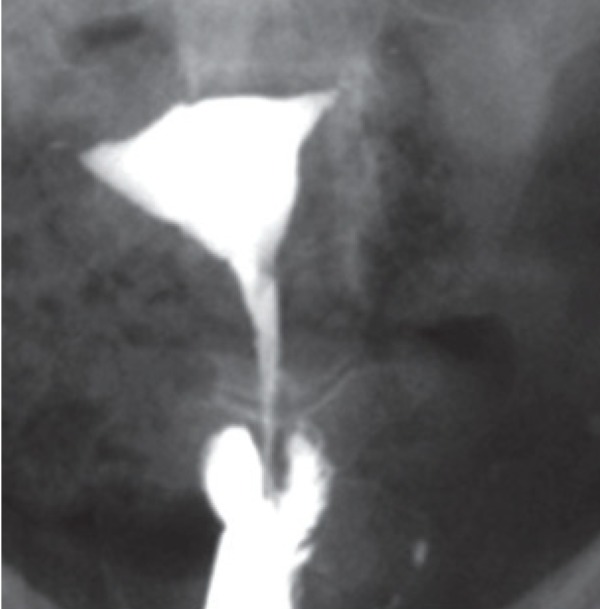
Infertility of a 27 year-old woman investigated. HSG demonstrates invagination of distal part of cervical canal within proximal part of cervix. In this patient proximal part of cervical canal is double but the distal portion is single and the patient has one cervical opening.

In the second case, the proximal part of the cervical canal was double, but the distal portion was single and one cervical opening was present (Fig .10).

**Fig.11:**
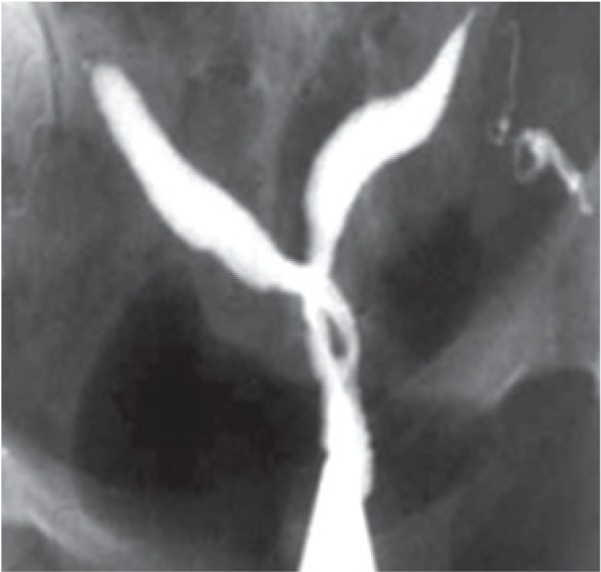
A 29- year-old woman with a history of 5 years of primary infertility. HSG represents a bicornuate uterus with communication at the level of isthmus, a septate cervix, and normal cervical os and vagina. The patient has no history of previous vaginal and cervical septum resection.

The third case was a bicornuate uterus with communication at the isthmic level, a septate cervix, and normal external cervical os and vagina (Fig .11). The patient had no history of any previous vaginal or cervical septum resection.

**Fig.12:**
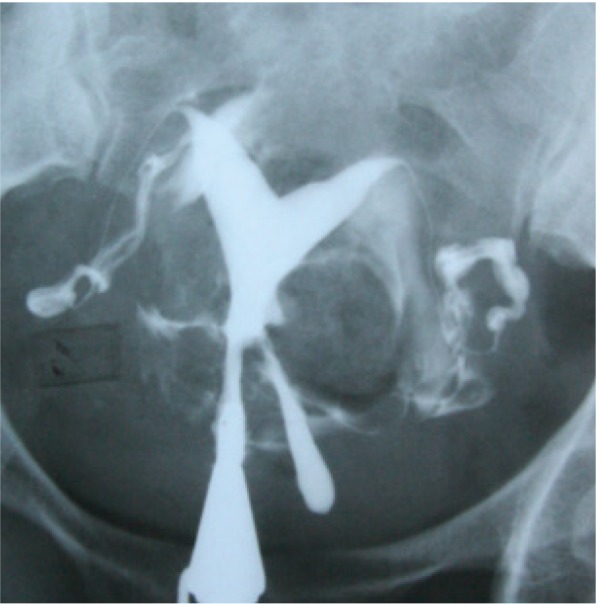
A 24-year-old woman with a history of 3 years infertility. HSG shows a septate uterus with a left blind hemivagina(atretica) and left renal agenesis (Herlyn-Werner- Wunderlich syndrome).

In the last case, HSG demonstrated a left blind hemivagina (atretica) with left renal agenesis (Herlyn-Werner- Wunderlich syndrome, Fig .12).

## Conclusion

Although congenital anomalies of the utero-cervix in the setting of MDAs are rare, the impact on a woman’s reproductive potential can be significant. Anomalies of the cervico-uterus are widely diagnosed by HSG. The diagnostic value of HSG in the detection of anomalies varies, depending on the type of malformation. Accurate diagnosis of these cases, especially the cases with any classification system, is important for optimal treatment and categorization of each anomaly.
